# Childhood trauma predicts antidepressant response in adults with major depression: data from the randomized international study to predict optimized treatment for depression

**DOI:** 10.1038/tp.2016.61

**Published:** 2016-05-03

**Authors:** L M Williams, C Debattista, A-M Duchemin, A F Schatzberg, C B Nemeroff

**Affiliations:** 1 Department of Psychiatry and Behavioral Sciences, Stanford University School of Medicine, Stanford, CA, USA; 2 Sierra-Pacific Mental Illness Research, Education, and Clinical Center, Veterans Affairs Palo Alto Health Care System, Palo Alto, CA, USA; 3 Department of Psychiatry, The Ohio State University College of Medicine, Columbus, OH, USA; 4 Department of Psychiatry and Behavioral Sciences, University of Miami Miller School of Medicine, Miami, FL, USA

## Abstract

Few reliable predictors indicate which depressed individuals respond to antidepressants. Several studies suggest that a history of early-life trauma predicts poorer response to antidepressant therapy but results are variable and limited in adults. The major goal of the present study was to evaluate the role of early-life trauma in predicting acute response outcomes to antidepressants in a large sample of well-characterized patients with major depressive disorder (MDD). The international Study to Predict Optimized Treatment for Depression (iSPOT-D) is a randomized clinical trial with enrollment from December 2008 to January 2012 at eight academic and nine private clinical settings in five countries. Patients (*n*=1008) meeting DSM-IV criteria for MDD and 336 matched healthy controls comprised the study sample. Six participants withdrew due to serious adverse events. Randomization was to 8 weeks of treatment with escitalopram, sertraline or venlafaxine with dosage adjusted by the participant's treating clinician per routine clinical practice. Exposure to 18 types of traumatic events before the age of 18 was assessed using the Early-Life Stress Questionnaire. Impact of early-life stressors—overall trauma ‘load' and specific type of abuse—on treatment outcomes measures: response: (⩾50% improvement on the 17-item Hamilton Rating Scale for Depression, HRSD_17_ or on the 16-item Quick Inventory of Depressive Symptomatology—Self-Rated, QIDS_SR_16_) and remission (score ⩽7 on the HRSD_17_ and ⩽5 on the QIDS_SR_16_). Trauma prevalence in MDD was compared with controls. Depressed participants were significantly more likely to report early-life stress than controls; 62.5% of MDD participants reported more than two traumatic events compared with 28.4% of controls. The higher rate of early-life trauma was most apparent for experiences of interpersonal violation (emotional, sexual and physical abuses). Abuse and notably abuse occurring at ⩽7 years of age predicted poorer outcomes after 8 weeks of antidepressants, across the three treatment arms. In addition, the abuses occurring between ages 4 and 7 years differentially predicted the poorest outcome following the treatment with sertraline. Specific types of early-life trauma, particularly physical, emotional and sexual abuse, especially when occurring at ⩽7 years of age are important moderators of subsequent response to antidepressant therapy for MDD.

## Introduction

Globally, 405 million people experience depression and depression is a leading contributor to disability and lost productivity.^1^ Currently, less than 50% of major depressive disorder (MDD) patients achieve remission following treatment with antidepressants. There is an urgent need to understand what factors determine who is likely to benefit, and who is not, from commonly used first-line treatments. Childhood trauma, particularly due to abuse and neglect, is among the most significant contributors to the subsequent development of MDD.^2, 3, 4, 5, 6, 7^ Despite this evidence, the rates of childhood trauma in outpatient depressed populations have not been well established and it remains unknown whether trauma predicts treatment outcomes in these outpatient populations. Thus, our aims were to establish the rates of trauma, overall and for individual types of trauma, in a large global sample of depressed outpatients and to assess whether exposure to these traumatic events is a general and/or differential predictor of outcomes following treatment with one of three commonly used antidepressants.

The exposure to significant traumatic stressors in childhood, including physical and sexual abuse and loss of parents, has been associated with an increased risk of depression.^3, 5^ Exposure to early trauma and abuse increase the likelihood of meeting criteria for a major depressive episode at any point in life,^6^ compared with the absence of exposure to trauma. Childhood abuse predicts poorer outcomes, including earlier-onset depression, a higher risk of recurrent depression, more severe course of illness and greater chronicity.^2, 8^

Several studies suggest that a history of pre-pubertal abuse or trauma also moderates response to treatment, however, with variable results.^8^ Nemeroff *et al.*^9^ studied 681 patients with chronic depression and their response to an antidepressant (nefazodone) or CBASP (cognitive behavioral analysis of psychotherapy), a form of cognitive behavioral therapy (CBT). Patients without a history of pre-pubertal stressor had an equivalent response to psychotherapy and nefazodone, and the best response to the combination treatment.^10^ However, patients who reported a history of parental loss before the age of 15, parental neglect or childhood physical or sexual abuse were significantly less likely to respond to pharmacotherapy alone. Furthermore, combination treatment did not substantially improve the outcome versus CBASP alone.

Some studies have suggested that specific types of abuse rather than a general history of trauma moderates treatment response. In the Treatment of Resistant Depression in Adolescents Study, 334 (ref. 11) adolescents who did not respond to a selective serotonin-reuptake inhibitor were randomized to receive 12 weeks of an alternative selective serotonin-reuptake inhibitor alone, or combined with CBT, or venlafaxine alone (a serotonin-norepinephrine reuptake inhibitor; SNRI) or with CBT. Those without a history of abuse did better on the combination treatment than on antidepressant monotherapy (62.8 vs 37.0%). Those with a history of sexual abuse responded equally to monotherapy and combination treatment (48.3 vs 42.3%), whereas those with a history of physical abuse responded substantially better to antidepressant monotherapy than combination treatment (52.4 vs 18.4%).

The Treatment for Adolescents with Depression Study^12^ compared the efficacy of 12 weeks fluoxetine, CBT, their combination and placebo in 427 adolescents with MDD, divided into four groups by their history: (1) no trauma; (2) trauma, no abuse; (3) physical abuse; and (4) sexual abuse. The no-trauma group responded to fluoxetine, with no effect of CBT over placebo. For teens with a history of trauma or physical abuse, no treatment was more effective than placebo. In the sexual abuse group, placebo appeared more effective than CBT. These findings are in contrast to Nemeroff *et al.*,^9^ in which psychotherapy appeared to be more effective than medication in depressed adults with a history of trauma or abuse. The difference in participant population (chronically depressed and adult vs children) could explain the discrepancy. Pediatric studies have generally suggested that a history of childhood trauma predicted a poorer response to psychotherapy.^13, 14, 15^ A recent meta-analysis^8^ concluded that a history of early-life stress predicted poorer response to pharmacotherapy and combination pharmacotherapy/psychotherapy, and a trend for poor response to psychotherapy as well.

The international Study to Predict Optimized Treatment in Depression (iSPOT-D) enrolled nonpsychotic MDD patients into a real-world practical effectiveness trial, primarily to identify pre-treatment characteristics as general or differential predictors of response to antidepressants that could change how practitioners select among antidepressant medications.^16^

In this study, we report on the effects of early-childhood trauma as a predictor of acute antidepressant treatment outcomes at 8 weeks post treatment in the 1008 depressed participants from iSPOT-D. Our aims were to test whether (i) the prevalence of early-life trauma differs in iSPOT-D MDD participants compared with matched healthy controls; (ii) the overall amount of ‘load' of early-life trauma is a general predictor of acute antidepressant treatment outcomes defined by response, remission and change in symptom severity; (iii) exposure to a specific type of abuse is a specific predictor of acute antidepressant treatment outcomes; and (iv) overall ‘load' of early-life trauma and exposure to a specific type of abuse are differential predictors of acute antidepressant treatment outcomes for different types of antidepressants.

## Materials and methods

### Sites and practitioners

The iSPOT-D is a multi-site, randomized practical clinical trial^9^ conducted at 17 sites in the United States, the Netherlands, Australia, New Zealand and South Africa. These sites include eight academic and nine private clinical settings. We refer to these as the ‘study management' sites because they manage recruitment and assessments. Most also act as the hub for a broader network of ‘care delivery' providers at community general practices and university general health centers, which prescribe and manage medication following usual clinical care.

### Design

The iSPOT-D is a multiple-phase, multi-site, real-world effectiveness study. The primary outcome is response at 8 weeks post baseline.^16^ The first phase enrolled 1008 MDD participants and 336 matched controls. The participants were assessed at the study sites at baseline and 8 weeks later.

### Participants

The study enrolled 1008 adults (aged 18–65 years) with a diagnosis of current, single-episode or recurrent nonpsychotic MDD and 336 controls with no diagnosis of mental disorder. Each site enrolled both MDD patients and healthy controls. The minimum number of participants per site was 30 and the maximum 200. The participants were enrolled between December 2008 and January 2012.

Psychiatric inclusion and exclusion criteria for MDD and controls were based on demographic information, medical and medication history, the Mini International Neuropsychiatric Interview, the 17-item Hamilton Rating Scale for Depression (HRSD_17_)^12^ to assess depressive severity (score ⩾16 for inclusion) and a urine toxicology screen. The exclusion criteria for all the participants included current or past diagnosis of psychosis, bipolar disorder, eating disorder, posttraumatic stress disorder and obsessive compulsive disorder as well as Axis II personality disorder. Known medical disease that might interfere with assessments was also exclusionary. In addition, for the control group, current or past history of MDD was exclusionary and for the MDD group, participants needed to meet inclusion criteria for MDD without psychosis (see Williams *et al.*^16^ for study design and Saveanu *et al.*^17^ for complete list of inclusion/exclusions). The medical history and adverse events for 1 year following the first visit both for controls and MDD participants were also recorded. The study was approved by each site's institutional review board and was carried out in accordance with the Declaration of Helsinki. All the participants provided written informed consent and received a stipend for their time and travel.

The participants with MDD had moderate-to-severe depression, with an average HRSD_17_ score of 21.9±4.1 (corresponding 16-item Quick Inventory of Depressive Symptomatology—Self-Rated (QIDS-SR_16_) score of 14.5±3.8). Almost 90% of the participants had recurrent MDD (average: five episodes). The average age at first episode was 22.9±12.0 years.

The sample size and power were determined as part of the protocol development.^16^ Our sample size was designed to provide statistical power of at least 89% power to detect small effects for predictors (odds ratio (OR) of 1.3 per standard deviation change in extent of trauma at the pre-treatment baseline) at an alpha level of *P*<0.05 and 94% power to detect medium effects (OR of 1.5). Further details about the participant sample are reported in Saveanu *et al.*^17^

### Study treatment

After pre-treatment assessments, MDD participants were randomized to receive escitalopram, sertraline or venlafaxine-extended release (XR). As outlined in the protocol for iSPOT-D,^16^ these three antidepressants were chosen because they are commonly used in practice in each country in which participating sites are located, are approved for MDD in the country of each participating site and have distinct pharmacological properties, which may enable the identification of moderators. The antidepressants were prescribed and doses adjusted by the participant's treating clinician according to routine clinical practice. Equal numbers of participants were randomly assigned to each treatment. The average treatment duration was 6.1±2.8 weeks at the time of follow-up assessment.

The average daily dose for each antidepressant was consistent with usual clinical prescribing practices (escitalopram: 12.3 mg per day; sertraline: 61.1 mg per day; venlafaxine-XR: 83.4 mg per day). Doses were in the lower end of recommended ranges compared with typical trials, consistent with the practical design of the trial which mirrored practice in primary care and community settings. Only 8% of participants used concomitant psychotropic medications, mostly anxiolytics (3.8%) and sleep aids (1.2%).

Adverse events were reported by 452 participants (44.8%), 88.3% likely related to the antidepressants. Six serious adverse events involved hospitalization, two for suicidal ideation/attempt, and led to early withdrawal. The adverse event rates did not significantly differ across treatment arms.^17^

### Treatment outcomes

The primary outcome was rate of response (⩾50% decrease in [HRSD_17_] scores from baseline to week 8) and remission (HRSD_17_ score ⩽7) to treatment. Secondary outcomes included response and remission by the QIDS-SR_16_ (response: ⩾50% decrease from baseline, remission: score ⩽5).^17, 18^ By the HRSD_17,_ 62.2% of participants met the criteria for response at week 8, and 45.4% were in remission. By the QIDS-SR_16_, 53.3% of participants responded and 37.6% were in remission (Saveanu *et al.*^17^). The response and remission rates did not significantly differ between the three treatment groups. We also assessed change in symptom severity on the HRSD_17_ and the QIDS-SR_16_ as a dimensional measure.

**Figure 1:**
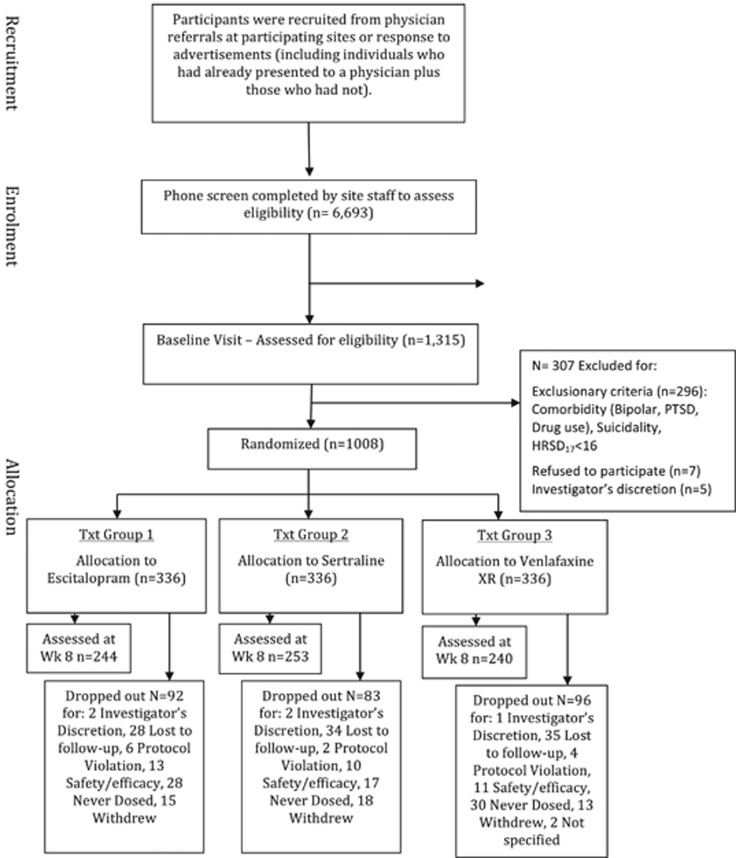
CONSORT chart. HRSD_17_, 17-item Hamilton Rating Scale for Depression; PTSD, posttraumatic stress disorder.

There was a 28.4% attrition rate that represented participants who were not medication adherent at week 8 or who withdrew from the study before week 8 (Figure 1).

### Assessment of childhood trauma

We used the Early-Life Stress Questionnaire (ELSQ)^19^ to assess the role of childhood trauma in relation to antidepressant outcomes. The ELSQ comprises 18 items, which assess exposure to specific traumatic events in the first 17 years of life (Table 1) and which are equivalent to the trauma items assessed by the Child Abuse and Trauma Scale.^20^ These events represent previously identified categories of trauma, including interpersonal violation (physical, psychological and sexual abuse, neglect, domestic violence, bullying), family breakup (divorce, separation, conflict), family health (death, life-threatening illness), personal health (hospitalization, life-threatening illness or injury), disaster/war (natural disaster, war), birth complications or adoption.^20, 21, 22, 23^ Each item is scored dichotomously for the presence/absence of exposure to each type of trauma. For each type of trauma endorsed as ‘present', participants also reported the age range in which the trauma occurred or first occurred (0–3, 4–7, 8–12 or 13–17 years of age). Previously, reports of childhood trauma using the ELSQ have been shown to correspond to registry-reported prevalence, and that reported trauma is predictive of severity of depressive symptoms.^19^

Extent of exposure to traumatic events was not found to differ across site (chi-square=10.954, df=6, *P*=0.090) or across country (chi-square=6.134, df=4, *P*=0.189).

### Statistical analysis

All analyses were conducted for the intention-to-treat sample. We established response and remission status based on the patients who completed treatment and the 8-week follow-up assessment (Figure 1). Responses to the ELSQ items were treated as categorical variables.

1. To test the hypothesis that prevalence of early-life trauma differs in MDD patients compared with controls, we used chi-square analysis and compared groups on each type of trauma.
2. We used logistic regression models to test whether overall trauma is a general predictor of acute treatment outcomes for response and remission defined by the HRSD_17_ and QIDS-SR_16_. Linear regression models were used to test prediction of dimensionally defined severity of symptoms at week 8, controlling for pre-treatment baseline severity.
3. We also used logistic and linear regression models to test whether abuse is a specific predictor of treatment outcomes. Given the focus on a particular trauma category, we used a corrected alpha level of 0.01.
4. We used logistic and linear regression models and included an interaction term for trauma by type of antidepressant to test whether overall trauma load or specific exposure to childhood abuse is a differential predictor of treatment outcomes for different types of antidepressants (escitalopram, sertraline, venlafaxine-XR).

In each regression model, clinical site, age and baseline level of depressive severity (on the HRSD_17_ or QIDS-SR_16_) were controlled for as covariates.

On the basis of the exponential beta values in the regression models, we generated the OR for each significant effect. The OR for interaction terms reflects how much greater/lower the likelihood of response/remission is in a multiplicative sense for treatment type.

## Results

### Prevalence of early-life trauma

To confirm the absence of mental disorder meeting criteria for DSM-IV, healthy controls were assessed by a MINI structured interview. They also completed all the assessments and had the same visit schedule as MDD participants. MDD participants were exposed to significantly more early-life stress than the healthy controls (Table 1): 62.5% of MDD participants reported more than two traumatic events compared with 28.4% of controls (Table 1). Most notably, MDD participants had a fourfold or higher rate of exposure to abuse (sexual, physical and emotional) than healthy controls (Table 1). Other types of interpersonal violation (neglect, witnessing domestic violence and bullying or rejection) were also significantly increased. More than 40% of the MDD participants reported experience of emotional abuse or bullying/rejection at school compared with less than 10% and 20%, respectively for controls. Having experienced parent divorce, separation from family, conflict with family or undergone major surgery or hospitalizations was also more frequent among MDD participants than controls, whereas other potential traumatic events like death in the family or natural disasters were not. Supplementary analyses showed that the group difference in ELSQ scores did not differ as a function of gender; hence, gender was not included as an additional variable in subsequent analyses.

### Overall presence of early-life trauma as a predictor of response and remission

Of 1008 participants randomized to treatment, 286 dropped out and 722 were assessed at week 8 (Figure 1). Overall presence of at least one early-life traumatic event was not a significant predictor of the percentage change in symptom severity from pre- to post-treatment, assessed by the HRSD_17_ and QIDS-SR_16_. Overall trauma was also not a significant predictor of response or remission defined categorically by the HRSD_17_ and QIDS-SR_16_.

### Specific contribution of abuse as a predictor of response and remission

**Figure 2:**
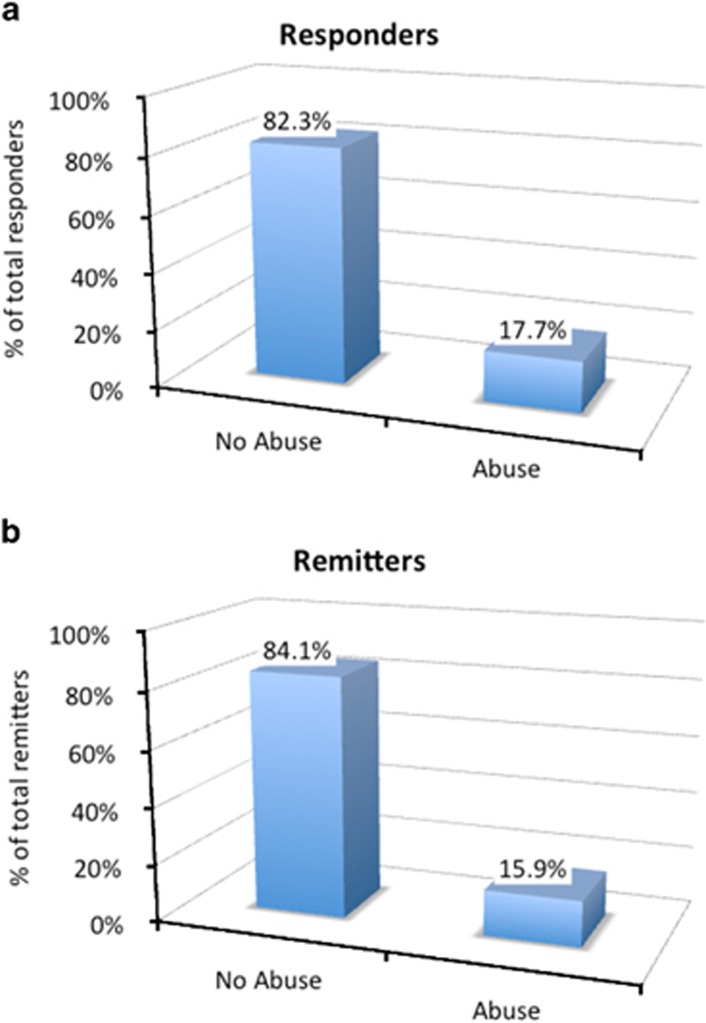
The effect of abuse at age 4–7 years on prediction for HRSD_17_ response and remission. HRSD_17_, 17-item Hamilton Rating Scale for Depression.

The experience of abuse was a significant predictor of both HRSD_17_-defined remission (chi-square=49.782, df=12, *P*<0.0001) and response (chi-square=72.769, df=12, *P*<0.0001). The experience of abuse at the age of 4 to 7 years contributed specifically and significantly to prediction for both HRSD_17_-defined response (*P*=0.034; OR=1.574) and remission (*P*=0.032; OR=1.606), indicating that participants were about 1.6 times less likely to achieve response or remission if exposed to abuse at this age (Figure 2).

Abuse occurring at age 4 to 7 years was also a predictor of percentage change in clinician-rated symptom severity assessed by the HRSD_17_ (F=2.787, *P*=0.007; specific contribution of abuse at 4 to 7 years; *t*=2.573, *P*=0.01; 50% mean symptom improvement in patients exposed to abuse at 4 to 7 years versus 58% in those not exposed). Similarly, abuse occurring at age 4 to 7 years was a predictor of dimensional change in self-reported symptom severity assessed by the QIDS-SR_16_ (F=6.507, *P*<0.0001; specific contribution of abuse at 4 to 7 years; *t*=2.242, *P*=0.025; 40% mean symptom improvement in patients exposed to abuse at 4 to 7 years versus 47% in those not exposed).

### Early-life abuse as a predictor of response and remission for specific types of treatment

Overall, the presence of early-life stressors occurring before 18 years of age did not interact with the type of treatment to predict response or remission. However, exposure to the specific trauma of abuse did interact with types of treatment to predict response and remission. Abuse included physical, sexual and emotional abuse items, which have been shown to form a cohesive factor.^19^

The experience of abuse predicted HRSD_17_-defined response according to type of treatment (omnibus chi-square=49.421, df=13, *P*<0.0001), specifically due to abuse at 4 to 7 years of age (*P*=0.043, interaction OR=1.225). The experience of abuse also predicted HRSD_17_-defined remission according to type of treatment (omnibus chi-square=75.136, df=13, *P*<0.0001), specifically due to abuse at 4 to 7 years of age (*P*=0.033, interaction OR=1.251). The experience of abuse also predicted QIDS-SR_16_-defined remission according to type of treatment (omnibus chi-square=36.322, df=13, *P*<0.0001), specifically due to abuse at 4 to 7 years of age (*P*=0.049, interaction OR=1.260). For sertraline, in particular, participants who were abused at this age showed significantly less improvement in both clinician (*P*<0.0001) and self-rated (*P*=0.007) symptoms, than with escitalopram and venlafaxine-XR treatments.

## Discussion

The iSPOT-D is the first real-world practical trial to assess the prevalence of childhood trauma and its impact on antidepressant outcomes in an international sample of outpatient treatment seekers with MDD. These outpatients were found to have a fourfold higher incidence of childhood abuse than their healthy peers, and a twofold higher incidence of early exposure to other traumatic events. The greater the exposure to abuse in particular, the less likely these depressed patients were to remit following treatment with one of the three commonly prescribed antidepressants. Our results suggest that remission rates may be especially low when abuse occurs during the very early period of 4–7 years of age, and following treatment with sertraline.

Overall, the higher rate of trauma observed in the present MDD sample is in line with registry and observational studies.^4, 9^ Thus, childhood trauma and especially abuse may contribute to the development of depression observed in routine practice in multiple outpatient settings spanning primary and specialist care settings. Our findings suggest that abuse in particular, and not overall exposure to traumatic events, predicts a lower rate of acute response and remission after antidepressant therapy. Sexual, physical and emotional abuse compared with other types of trauma (such as death of a parent/sibling, personal life-threatening illness/injury, or disaster), may have a specific impact on the neurobiological mechanisms of non-response to treatment. Neuroimaging studies suggest that there may be a differential effect of childhood sexual abuse on the subsequent functioning of emotional brain circuits in adulthood depression.^24^ Childhood abuse has also been associated with a greater sensitivity to stress,^4^ cognitive impairment,^25^ alterations in brain morphometry^26, 27, 28, 29, 30^ and immune and metabolic abnormalities^31^ that may impact the course of depression and capacity to respond to antidepressants. It is also possible that abuse may recur and that it is the recurrence of the trauma that produces poor treatment outcomes.

In addition to the type of stressor, our results suggest that there is a critical period (4 to 7 years) in which childhood trauma occurs and has the most significant impact on subsequent poor response to antidepressants in adulthood. There is a rapidly growing body of work to suggest that gene polymorphisms and epigenetic modifications interact with childhood trauma to exert their effect on risk for depression,^32, 33, 34^ and that this effect is greatest at critical neurodevelopmental periods.^7, 35^ This evidence is consistent with the view that the extent of brain plasticity varies during development, such that trauma occurring during critical neurodevelopmental periods may alter brain morphometry, circuit function, endocrine regulation, immune function and subsequent physiologic reactions to stress in an enduring way.^28, 29, 36, 37^

Abuse occurring at age 4 to 7 years was associated with significantly poorer outcomes following the treatment with sertraline compared with the other selective serotonin-reuptake inhibitor escitalopram and the SNRI venlafaxine-XR. The participants who were abused at this age showed significantly less improvement in both clinician and self-rated symptoms following 8 weeks treatment with sertraline. Sertraline, in contrast to other serotonin-reuptake antidepressants, has an additional relatively specific effect on inhibiting dopamine.^38^ There is some evidence that subgroups of patients are also characterized by dopamine circuit dysfunction^39^ and number of traumatic events has been associated with higher ventral striatal dopamine response to amphetamine.^40^ Although speculative, these lines of evidence suggest that a possible dopamine-related mechanism might contribute to our specific observation of especially poor outcomes following sertraline in those exposed to early abuse.

### Clinical translational significance

Here, we provide evidence from a well-powered study that outpatients with MDD have a fourfold higher incidence of childhood abuse than their healthy peers, and a twofold higher incidence of early exposure to other traumatic events. The greater the exposure to trauma, the less likely these depressed patients were to remit following antidepressant response. Thus, the translational significance of these findings is that in routine clinical management of depression it may be important to screen for childhood trauma to identify those patients that may not benefit from standard first-line antidepressants and may require additional therapy to more directly address the impact of trauma.

### Limitations

The results should be interpreted within the context of several potential limitations of the study. First, there may be limitations due to sampling. We had a higher percentage of males relative to females than reported in previous studies such as STAR*D. This difference might be because we used a practical biomarker trial design and that males were more likely to report depression and trauma in this context. However, because women have previously been more likely to report trauma than men,^41^ future studies are required to establish the generalizability of our findings.

The study might also be limited due to the assessment of early-childhood trauma by the ELSQ. The scale is dependent on the recollection of the subject, and age 4–7 years may be the earliest reliable recollection rather than representing only a specific window of vulnerability. However, this limitation would not account for the lack of specific effect of abuse experienced after 7 years of age. Retrospective reports of childhood trauma, such as those assessed by the ELSQ, may have some bias but also tend to err on the side of false negatives rather than false positives.^42^ The prevalence of trauma reported retrospectively using the ELSQ is comparable with prevalence rates from archival records^19^ suggesting that such retrospective reports are also reliable. Nonetheless, it would be important to establish the reproducibility of our findings using additional and independent measures. Design limitations of this study include the reliance on only three first-step antidepressants, though they are commonly used in practice. Doses were lower than midrange of the recommended range, perhaps because the response and remission rates were large enough that further dose escalation was not warranted in many patients. Furthermore, as dose ranges were reflective of usual management practices and since primary care physicians prescribe about two-thirds of antidepressants,^43^ these ranges are an appropriate starting point for identifying predictors of outcome in real-world management settings. Although the present sample is well powered, we recognize that the findings are limited to this sample and that it would be beneficial to seek to replicate the findings in another sample. The iSPOT-D is designed to address future replication in a second cohort of a similar sample size.

## Conclusion

Here we provide a systematic investigation to show that there is a fourfold increase in the rate of childhood trauma, especially physical, emotional and sexual abuse,^35^ in outpatients with depression. Abuse occurring before the age of 7 years predicts substantially poorer response and remission outcomes for commonly prescribed antidepressants. These outcomes may be poorest for sertraline. These results suggest that it is important to assess for childhood trauma in the outpatient management of depression, and to consider alternative or supplemental treatments for patients with a trauma history.

## Figures and Tables

**Table 1:** Prevalence of early-childhood trauma in depressed patients and in healthy controls, according to the category and type of trauma

*Category and type of trauma (ELSQ items: yes/no questions)*	*% Prevalence of trauma*		*Group difference*	
	*MDD* N=*1008* n*(%)*	*Control* N=*336* n*(%)*	χ^*2*^	P*-value*
*Abuse/interpersonal violation*				
Were you physically abused?	219 (21.7)	18 (5.4)	41.925	<0.001
Were you sexually abused?	164 (16.3)	18 (5.4)	27.758	<0.001
Were you emotionally abused?	433 (43.0)	31 (9.2)	113.225	<0.001
Did you experience extreme poverty or neglect?	186 (18.5)	15 (4.5)	36.118	<0.001
Did you witness domestic violence within your family?	326 (32.3)	49 (14.6)	36.560	<0.001
Did you experience sustained bullying or rejection by schoolmates?	452 (44.8)	60 (17.9)	82.231	<0.001
				
*Family breakup*				
Did your parents divorce or separate?	408 (40.5)	103 (30.7)	11.894	0.02
Were you separated for a long period from a parent, brother or sister?	439 (43.6)	61 (18.2)	30.035	<0.001
Was there sustained conflict within your family?	516 (51.2)	112 (33.3)	70.875	<0.001
				
*Family health/death*				
Did one of your parents, a brother or sister die?	92 (9.1)	26 (7.7)	5.403	0.25
Did one of your parents, a brother or sister experience a life-threatening illness?	168 (16.7)	36 (10.7)	6.818	0.15
				
*Personal health*				
Did you undergo major surgery or repeated hospitalization?	168 (16.7)	32 (9.5)	9.559	0.05
Did you experience a life-threatening illness or injury?	100 (9.9)	27 (8.0)	5.227	0.27
				
*Disaster/war*				
Did you witness first-hand a natural disaster such as earthquake, flood or fire?	121 (12.0)	39 (11.6)	4.229	0.38
Did you witness warfare?	29 (2.9)	7 (2.1)	0.357	0.55
				
*Other traumatic events*				
Were you born prematurely, or experience other birth complications?	132 (13.1)	29 (8.6)	3.895	0.048
Were you adopted?	43 (4.3)	8 (2.4)	3.399	0.49
Was your house destroyed by fire or other means?	43 (4.3)	14 (4.2)	2.711	0.61

Abbreviations: ELSQ, Early-Life Stress Questionnaire; MDD, major depressive disorder.

## References

[bib1] Whiteford HA, Degenhardt L, Rehm J, Baxter AJ, Ferrari AJ, Erskine HE et al. Global burden of disease attributable to mental and substance use disorders: findings from the Global Burden of Disease Study 2010. Lancet 2013; 382: 1575–1586. 23993280 10.1016/S0140-6736(13)61611-6

[bib2] Bernet CZ, Stein MB. Relationship of childhood maltreatment to the onset and course of major depression in adulthood. Depress Anxiety 1999; 9: 169–174. 10431682

[bib3] Dube SR, Anda RF, Whitfield CL, Brown DW, Felitti VJ, Dong M et al. Long-term consequences of childhood sexual abuse by gender of victim. Am J Prev Med 2005; 28: 430–438. 15894146 10.1016/j.amepre.2005.01.015

[bib4] Hammen C, Henry R, Daley SE. Depression and sensitization to stressors among young women as a function of childhood adversity. J Consult Clin Psychol 2000; 68: 782–787. 11068964

[bib5] Kendler KS, Kessler RC, Walters EE, MacLean C, Neale MC, Heath AC et al. Stressful life events, genetic liability, and onset of an episode of major depression in women. Am J Psychiatry 1995; 152: 833–842. 7755111 10.1176/ajp.152.6.833

[bib6] Kessler RC, Davis CG, Kendler KS. Childhood adversity and adult psychiatric disorder in the US National Comorbidity Survey. Psychol Med 1997; 27: 1101–1119. 9300515 10.1017/s0033291797005588

[bib7] Nemeroff CB, Binder E. The preeminent role of childhood abuse and neglect in vulnerability to major psychiatric disorders: toward elucidating the underlying neurobiological mechanisms. J Am Acad Child Adolesc Psychiatry 2014; 53: 395–397. 24655648 10.1016/j.jaac.2014.02.004

[bib8] Nanni V, Uher R, Danese A. Childhood maltreatment predicts unfavorable course of illness and treatment outcome in depression: a meta-analysis. Am J Psychiatry 2012; 169: 141–151. 22420036 10.1176/appi.ajp.2011.11020335

[bib9] Nemeroff CB, Heim CM, Thase ME, Klein DN, Rush AJ, Schatzberg AF et al. Differential responses to psychotherapy versus pharmacotherapy in patients with chronic forms of major depression and childhood trauma. Proc Natl Acad Sci USA 2003; 100: 14293–14296. 14615578 10.1073/pnas.2336126100 PMC283585

[bib10] Keller MB, McCullough JP, Klein DN, Arnow B, Dunner DL, Gelenberg AJ et al. A comparison of nefazodone, the cognitive behavioral-analysis system of psychotherapy, and their combination for the treatment of chronic depression. N Engl J Med 2000; 342: 1462–1470. 10816183 10.1056/NEJM200005183422001

[bib11] Shamseddeen W, Asarnow JR, Clarke G, Vitiello B, Wagner KD, Birmaher B et al. Impact of physical and sexual abuse on treatment response in the Treatment of Resistant Depression in Adolescent Study (TORDIA). J Am Acad Child Adolesc Psychiatry 2011; 50: 293–301. 21334569 10.1016/j.jaac.2010.11.019 PMC3073648

[bib12] Lewis CC, Simons AD, Nguyen LJ, Murakami JL, Reid MW, Silva SG et al. Impact of childhood trauma on treatment outcome in the Treatment for Adolescents with Depression Study (TADS). J Am Acad Child Adolesc Psychiatry 2010; 49: 132–140. 20215935 10.1097/00004583-201002000-00007

[bib13] Asarnow JR, Emslie G, Clarke G, Wagner KD, Spirito A, Vitiello B et al. Treatment of selective serotonin reuptake inhibitor-resistant depression in adolescents: predictors and moderators of treatment response. J Am Acad Child Adolesc Psychiatry 2009; 48: 330–339. 19182688 10.1097/CHI.0b013e3181977476 PMC2754157

[bib14] Barbe RP, Bridge JA, Birmaher B, Kolko DJ, Brent DA. Lifetime history of sexual abuse, clinical presentation, and outcome in a clinical trial for adolescent depression. J Clin Psychiatry 2004; 65: 77–83. 14744173 10.4088/jcp.v65n0113

[bib15] Shirk SR, Karver M. Prediction of treatment outcome from relationship variables in child and adolescent therapy: a meta-analytic review. J Consult Clin Psychol 2003; 71: 452–464. 12795570 10.1037/0022-006x.71.3.452

[bib16] Williams LM, Rush AJ, Koslow SH et al. International Study to Predict Optimized Treatment for Depression (iSPOT-D), a randomized clinical trial: rationale and protocol. Trials 2011; 12: 4. 21208417 10.1186/1745-6215-12-4 PMC3036635

[bib17] Saveanu R, Etkin A, Duchemin A-E, Goldstein-Piekarski A, Gyurak A, Debattista C et al. The International Study to Predict Optimized Treatment in Depression (iSPOT-D): outcomes from the acute phase of antidepressant treatment. J Psychiatric Res 2015; 61: 1–12. 10.1016/j.jpsychires.2014.12.018 25586212

[bib18] Rush AJ, Trivedi MH, Ibrahim HM, Carmody TJ, Arnow B, Klein DN et al. The 16-Item Quick Inventory of Depressive Symptomatology (QIDS), clinician rating (QIDS-C), and self-report (QIDS-SR): a psychometric evaluation in patients with chronic major depression. Biol Psychiatry 2003; 54: 573–583. 12946886 10.1016/s0006-3223(02)01866-8

[bib19] Trivedi MH, Rush AJ, Ibrahim HM, Carmody TJ, Biggs MM, Suppes T et al. The Inventory of Depressive Symptomatology, Clinician Rating (IDS-C) and Self-Report (IDS-SR), and the Quick Inventory of Depressive Symptomatology, Clinician Rating (QIDS-C) and Self-Report (QIDS-SR) in public sector patients with mood disorders: a psychometric evaluation. Psychol Med 2004; 34: 73–82. 14971628 10.1017/s0033291703001107

[bib20] Chu DA, Williams LM, Harris AW, Bryant RA, Gatt JM. Early life trauma predicts self-reported levels of depressive and anxiety symptoms in nonclinical community adults: Relative contributions of early life stressor types and adult trauma exposure. J Psychiatr Res 2013; 47: 23–32. 23020924 10.1016/j.jpsychires.2012.08.006

[bib21] Sanders B, Becker-Lausen E. The measurement of psychological maltreatment: early data on the Child Abuse and Trauma Scale. Child Abuse Negl 1995; 19: 315–323. 9278731 10.1016/s0145-2134(94)00131-6

[bib22] Becker-Lausen E, Sanders B, Chinsky JM. Mediation of abusive childhood experiences: depression, dissociation, and negative life outcomes. Am J Orthopsychiatry 1995; 65: 560–573. 8561189 10.1037/h0079670

[bib23] Kent A, Waller G. The impact of childhood emotional abuse: an extension of the Child Abuse and Trauma Scale. Child Abuse Negl 1998; 22: 393–399. 9631251 10.1016/s0145-2134(98)00007-6

[bib24] Mark TL, Levit KR, Buck JA. Psychotropic drug prescriptions by medical specialty. Psychiatr Serv 2009; 60: 1167. 19723729 10.1176/ps.2009.60.9.1167

[bib25] Majer M, Nater UM, Lin JM, Capuron L, Reeves WC. Association of childhood trauma with cognitive function in healthy adults: a pilot study. BMC Neurol 2010; 10: 61. 20630071 10.1186/1471-2377-10-61 PMC2910667

[bib26] Heim C, Shugart M, Craighead WE, Nemeroff CB. Neurobiological and psychiatric consequences of child abuse and neglect. Dev Psychobiol 2010; 52: 671–690. 20882586 10.1002/dev.20494

[bib27] Heim CM, Mayberg HS, Mletzko T, Nemeroff CB, Pruessner JC. Decreased cortical representation of genital somatosensory field after childhood sexual abuse. Am J Psychiatry 2013; 170: 616–623. 23732967 10.1176/appi.ajp.2013.12070950

[bib28] Huang LT. Early-life stress impacts the developing hippocampus and primes seizure occurrence: cellular, molecular, and epigenetic mechanisms. Front Mol Neurosci 2014; 7: 8. 24574961 10.3389/fnmol.2014.00008 PMC3918912

[bib29] Lupien SJ, McEwen BS, Gunnar MR, Heim C. Effects of stress throughout the lifespan on the brain, behaviour and cognition. Nat Rev Neurosci J 2009; 10: 434–445. 10.1038/nrn2639 19401723

[bib30] McCrory E, De Brito SA, Viding E. The link between child abuse and psychopathology: a review of neurobiological and genetic research. J R Soc Med 2012; 105: 151–156. 22532655 10.1258/jrsm.2011.110222 PMC3343716

[bib31] Danese A, Moffitt TE, Harrington H, Milne BJ, Polanczyk G, Pariante CM et al. Adverse childhood experiences and adult risk factors for age-related disease: depression, inflammation, and clustering of metabolic risk markers. Arch Pediatr Adolesc Med 2009; 163: 1135–1143. 19996051 10.1001/archpediatrics.2009.214 PMC3560401

[bib32] Skokauskas N, Carballedo A, Fagan A, Frodl T. The role of sexual abuse on functional neuroimaging markers associated with major depressive disorder. World J Biol Psychiatry 2015; 16: 513–520. 26114449 10.3109/15622975.2015.1048723

[bib33] Gatt JM, Nemeroff CB, Schofield PR, Paul RH, Clark CR, Gordon E et al. Early life stress combined with serotonin 3A receptor and brain-derived neurotrophic factor valine 66 to methionine genotypes impacts emotional brain and arousal correlates of risk for depression. Biol Psychiatry 2010; 68: 818–824. 20728877 10.1016/j.biopsych.2010.06.025

[bib34] Williams LM, Gatt JM, Schofield PR, Olivieri G, Peduto A, Gordon E. ‘Negativity bias' in risk for depression and anxiety: brain-body fear circuitry correlates, 5-HTT-LPR and early life stress. Neuroimage 2009; 47: 804–814. 19446647 10.1016/j.neuroimage.2009.05.009

[bib35] Heim C, Binder EB. Current research trends in early life stress and depression: review of human studies on sensitive periods, gene-environment interactions, and epigenetics. Exp Neurol 2012; 233: 102–111. 22101006 10.1016/j.expneurol.2011.10.032

[bib36] McGee RA, Wolfe DA, Yuen SA, Wilson SK, Carnochan J. The measurement of maltreatment: a comparison of approaches. Child Abuse Negl 1995; 19: 233–249. 7780784 10.1016/0145-2134(94)00119-f

[bib37] Kaffman A, Meaney MJ. Neurodevelopmental sequelae of postnatal maternal care in rodents: clinical and research implications of molecular insights. J Child Psychol Psychiatry 2007; 48: 224–244. 17355397 10.1111/j.1469-7610.2007.01730.x

[bib38] Gatt JM, Nemeroff CB, Dobson-Stone C, Paul RH, Bryant RA, Schofield PR et al. Interactions between BDNF Val66Met polymorphisms and early life stress predict brain and arousal pathways to syndromal depression and anxiety. Mol Psychiatry 2009; 14: 681–695. 19153574 10.1038/mp.2008.143

[bib39] McEwen BS. Brain on stress: how the social environment gets under the skin. Proc Natl Acad Sci USA 2012; 109(Suppl 2): 17180–17185. 23045648 10.1073/pnas.1121254109 PMC3477378

[bib40] Camardese G, Di Giuda D, Di Nicola M, Cocciolillo F, Giordano A, Janiri L et al. Imaging studies on dopamine transporter and depression: a review of literature and suggestions for future research. J Psychiatr Res 2014; 51: 7–18. 24433847 10.1016/j.jpsychires.2013.12.006

[bib41] Oswald LM, Wand GS, Kuwabara H, Wong DF, Zhu S, Brasic JR. History of childhood adversity is positively associated with ventral striatal dopamine responses to amphetamine. Psychopharmacology 2014; 231: 2417–2433. 24448898 10.1007/s00213-013-3407-z PMC4040334

[bib42] Baum N, Rahav G, Sharon M. Heightened susceptibility to secondary traumatization: a meta-analysis of gender differences. Am J Orthopsychiatry 2014; 84: 111–122. 24826927 10.1037/h0099383

[bib43] Hardt J, Rutter M. Validity of adult retrospective reports of adverse childhood experiences: review of the evidence. J Child Psychol Psychiatry 2004; 45: 260–273. 14982240 10.1111/j.1469-7610.2004.00218.x

